# On the Corner of Models and Cure: Gene Editing in Cystic Fibrosis

**DOI:** 10.3389/fphar.2021.662110

**Published:** 2021-04-27

**Authors:** Marjolein Ensinck, Angélique Mottais, Claire Detry, Teresinha Leal, Marianne S. Carlon

**Affiliations:** ^1^ Molecular Virology and Gene Therapy, Department of Pharmaceutical and Pharmacological Sciences, KU Leuven, Leuven, Belgium; ^2^ Institut de Recherche Expérimentale et Clinique, Louvain Centre for Toxicology and Applied Pharmacology, Université Catholique de Louvain, Brussels, Belgium

**Keywords:** cystic fibrosis, CFTR, gene therapy, gene editing, CF cell models, CF animal models, humanized CF animals

## Abstract

Cystic fibrosis (CF) is a severe genetic disease for which curative treatment is still lacking. Next generation biotechnologies and more efficient cell-based and *in vivo* disease models are accelerating the development of novel therapies for CF. Gene editing tools, like CRISPR-based systems, can be used to make targeted modifications in the genome, allowing to correct mutations directly in the Cystic Fibrosis Transmembrane conductance Regulator (*CFTR*) gene. Alternatively, with these tools more relevant disease models can be generated, which in turn will be invaluable to evaluate novel gene editing-based therapies for CF. This critical review offers a comprehensive description of currently available tools for genome editing, and the cell and animal models which are available to evaluate them. Next, we will give an extensive overview of proof-of-concept applications of gene editing in the field of CF. Finally, we will touch upon the challenges that need to be addressed before these proof-of-concept studies can be translated towards a therapy for people with CF.

## Introduction

Cystic fibrosis (CF) is a rare genetic disease affecting approximately 80,000 people worldwide (Jackson and Goss, 2018). More than 2,100 mutations have been identified in the *CFTR* (Cystic Fibrosis Transmembrane conductance Regulator) gene (www.genet.sickkids.on.ca), of which currently 360 reported as disease-causing (www.CFTR2.org). The gene encodes a chloride/bicarbonate channel that plays an essential role in the fluid and electrolyte balance across secretory epithelia. Mutations in *CFTR* can disrupt the function of the protein through a variety of mechanisms, ranging from reduced or absent protein synthesis to normal apical expression of proteins with defective chloride and bicarbonate gating or conductance (De Boeck and Amaral, 2016; Veit et al., 2016). The protein loss-of-function results in dehydration of the surface liquid lining the epithelia of various organs, such as the airways, the gastrointestinal and reproductive tracts and the sweat glands. The respiratory phenotype is characterized by recurrent cycles of mucostasis, inflammation and infection that progressively destroys the lung architecture leading to respiratory failure and death (Boon et al., 2016). As CFTR is expressed in a variety of exocrine epithelia, with some of the disease symptoms already starting just after birth, or even *in utero*, this multisystemic disease requires an early, multidisciplinary management (reviewed in (Carlon et al., 2017)). Despite better symptomatic care, the median age at death remains in the early forties (Wanyama and Thomas, 2018). The development of effective CFTR modulator drugs which either improve CFTR processing (correctors) or channel gating (potentiators), brings the prospect of further increasing life quality and expectancy (Ramsey et al., 2011; Wainwright et al., 2015; Taylor-Cousar et al., 2017; Keating et al., 2018; Heijerman et al., 2019; Middleton et al., 2019). Currently, four modulator regimens are approved, consisting of Kalydeco™, a potentiator monotherapy, and three combination therapies consisting of one potentiator with one or two correctors with distinct mode of action (Orkambi™, Symdeko™, and Trikafta™). Together, they can functionally correct approximately 184 different CF-causing mutations, accounting for 90% of individuals with CF. Clinical benefits range from significant increases in lung function, to modest or even absent clinical improvements. Indeed, even the highly effective Trikafta™ showed variable clinical responses in patients with at least one F508del (c.1521–1523delCTT) allele (Heijerman et al., 2019; Middleton et al., 2019), likely influenced by the individual genetic and environmental background. Besides that, adverse events have been reported, such as chest tightness, diarrhea and drug-drug interactions, which in the context of a life-long intake is not desirable (Wainwright et al., 2015; Talamo Guevara and McColley, 2017; Guimbellot et al., 2018; Burgel et al., 2020). For Kalydeco™, the clinical benefits have been monitored over many years already, showing that after a significant increase in lung function at treatment onset, CF lung disease continues to worsen, although at a slower pace (Volkova et al., 2020). Finally, therapeutic options that tackle the CFTR defect are still missing for approximately 91% of *CFTR* mutations covering 10% of people with CF (PwCF). These carry two minimal function mutations, comprising frameshifts, large insertions and deletions (indels), nonsense and drug-refractory missense mutations. While pharmacological advances have greatly improved life quality of PwCF, and very likely will increase life expectancy as well, none of these treatments provide a cure.

Since the discovery of the *CFTR* gene 30 years ago (Kerem et al., 1989; Riordan et al., 1989; Rommens et al., 1989), scientists have been trying to bring gene therapy to PwCF. In contrast to CFTR modulators, gene therapy aims to restore the defect at the DNA level, thus providing a long-term correction. Traditionally, gene therapy efforts were focused on delivering a copy of the *CFTR* cDNA, providing cells with mutation agnostic means to produce wild type (WT) CFTR protein (Vidovic et al., 2016; Alton et al., 2017). Clinical trials investigating different gene addition strategies have not resulted in meaningful clinical benefit (reviewed in (Sondhi et al., 2017)). However, successes in other genetic diseases leading to the market approval of gene therapeutic agents, such as for retinal dystrophy (Russell et al., 2017), spinal muscular atrophy type 1 (Mendell et al., 2017), and *β*-thalassemia (Harrison, 2019), have in part revived the interest for CF gene therapy research. Indeed, the current priority of the CF community is to develop a causal therapy and preferably even a cure for all individuals with CF, as underscored by the Cystic Fibrosis Foundation’s “Path to a Cure” mission statement (CFF, 2019).

In this review, we will discuss the most recent advances in gene editing with respect to both the development of human-relevant disease models of CF as well as proof of concept studies that have shown the feasibility and safety of correcting a variety of *CFTR* mutations. Furthermore, we discuss some of the hurdles and open questions which need to be solved, to allow further progress of these promising but early gene editing studies. Finally, we discuss possible strategies that would facilitate the further development of an *in vivo* or *ex vivo* gene therapeutic approach toward its future clinical application.

## How to Edit the Genome: Gene Editing Tools

Advances in gene editing tools have made targeted genome modifications widely available. Zinc finger nucleases (ZFN), transcription activator-like effector nucleases (TALEN) and in particular CRISPR (clustered regularly interspaced short palindromic repeats) systems have opened up a seemingly endless variety of possible alterations to be introduced into the genome. Correcting mutations at their endogenous loci heralds a new era of personalized medicine and treatment of monogenetic diseases, by correcting the underlying cause of the disease. Alternatively, mutations can be introduced in a targeted manner, allowing elucidation of disease mechanisms and testing of potential therapies. Especially when a monogenic disorder is caused by many, exceedingly rare mutations, as is the case for CF, introducing mutations into relevant cell and animal models provides a unique opportunity to study these mutations when primary material is not easily obtained. Besides, samples are usually not available in a homozygous manner, due to the low prevalence of each individual mutation, and analysis of compound heterozygous samples is complicated by the presence of another mutation on the other allele. Different editing strategies however, will be required to cover all base conversions and indels that cause CF, both for the development of gene editing-based therapies as well as new CF model systems. In the following part, we will briefly discuss the different tools available for gene-editing. Proof-of-concept applications of these tools will be discussed in the section *Gene Editing to Correct Mutations in CFTR*.

### ZFN, TALEN, and Cas Nucleases

Programable nucleases like ZFN, TALEN, and CRISPR associated (Cas) nucleases, are all used to generate targeted double strand breaks (DSBs) in the genome. Editing at these sites relies on repair of the DSB by cellular DNA damage repair mechanisms. Most commonly, DSBs are repaired by a process called non-homologous end joining (NHEJ) where the two ends are re-ligated (Guirouilh-Barbat et al., 2004). As this process is imperfect, some bases can get deleted or inserted, creating indels. This can result in frameshifts disrupting the coding sequence, effectively knocking out proteins, which has been useful to study the function of genes. In the era of personalized medicine however, models harboring specific mutations are often preferred as they allow to study the disease-causing mutations in detail as well as test potential rescue strategies for these mutations. The therapeutic potential of NHEJ is rather limited since it can only be used to correct defects in certain cases e.g., gain-of-function mutations and mutations resulting in cryptic splice sites in introns where indels are tolerated. Many genetic diseases, however, including CF, are caused by loss-of-function mutations which cannot be repaired by NHEJ, with the exception of a couple of intronic splicing mutations. Introduction of specific edits relies on the alternative DNA damage repair mechanism: homology directed repair (HDR). A template, a DNA molecule containing the desired edit and homology arms on both sides, is used by the HDR machinery to repair the DSB and introduce the edit to the genome (reviewed in (Hsu et al., 2014)). HDR however is less efficient compared to NHEJ and is only active in dividing cells, as it normally protects cells against DNA damage during DNA replication. Therefore, its use in many adult cell types is limited. For a schematic overview of the two repair pathways and their possible applications, we refer to (Pranke et al., 2019; Maule et al., 2020).

The different nuclease-based gene editing tools differ mainly in their nuclease and the way specific DNA sequences are recognized. ZFNs were the first available “molecular scissors” and were built using multiple zinc finger domains (that each recognize a three bp DNA motif) fused to the FokI nuclease (reviewed in (Urnov et al., 2010)). The DNA is cleaved when two ZFNs are recruited to a target sequence, resulting in the dimerization of the FokI nuclease domains. Similarly, TALENs use transcription activator-like effectors (TALEs) rather than zinc fingers to recognize specific DNA sequences, also fused to the FokI nuclease (reviewed in (Joung and Sander, 2013)). Both ZFNs and TALENs require custom protein design for each target, in contrast to CRISPR-Cas based systems. Cas nucleases were first described as a gene editing tool in 2012 (Gasiunas et al., 2012; Jinek et al., 2012) and CRISPR-Cas gene editing rapidly became one of the cornerstones of modern biomedical research. It was hardly surprising that Jennifer Doudna and Emmanuelle Charpentier were awarded the 2020 Nobel prize in Chemistry for their pioneering work in the development of this highly versatile editing tool (Ledford and Callaway, 2020). The most used and best studied Cas protein is derived from *Streptococcus pyogenes* (SpCas9) and finds its DNA target using a short RNA, the single guide RNA (sgRNA), which consists of a 20 nucleotide spacer and a scaffold which it uses to interact with the Cas9 protein. Once the Cas9-sgRNA complex is formed, it will scan the DNA for sequences (the protospacer) that match the sgRNA-spacer motif at sites where protospacer adjacent motifs (PAM, NGG for SpCas9) are present, and if a match is found, the nuclease activity of Cas9 is activated. Cas9 however has some leniency toward mismatches between spacer and protospacer, which might result in unwanted off-targets, requiring further investigation, in particular with regard to therapeutic applications. A combination of *in silico* off-target prediction tools as well as experimental set-ups e.g., GUIDE-seq (Tsai et al., 2015), allow to identify (potential) off-target sites, which can then be evaluated by deep sequencing. For an overview of different Cas proteins and optimizations, for example to increase the number of targetable sites or improve efficacy or specificity, we refer to (Anzalone et al., 2020; Makarova et al., 2020; Maule et al., 2021).

### Base Editing

Although CRISPR-Cas editing itself was still relatively new, CRISPR-Cas derived techniques rapidly emerged, one of which is base editing. One of the largest assets of base editors is the ability to perform targeted base modifications without introducing DSBs into the genome. Besides, base editing does not rely on HDR and can therefore also be performed in cells that are not actively dividing. Base editors employ a Cas9n (nickase) in which one of the nuclease sites has been knocked out (D10A) fused to a deaminase. For cytosine base editors (CBE), this is a rAPOBEC deaminase which will deaminate cytosines after the Cas9n-sgRNA complex has guided the deaminase to the target (Komor et al., 2016). Deamination results in uracil which is repaired to thymine while the Cas9n will nick the non-edited strand, pushing DNA damage repair toward incorporating the edit. Inhibition of the base excision repair (BER) pathway by adding an uracil glycosylase inhibitor (UGI) to the CBE, further increased C-to-T editing efficiencies. Alternatively, instead of inhibiting the uracil DNA glycosylase (UNG) which initiates BER, promoting it often results in uracil to guanine repair, effectively creating C-to-G base editors (CGBE) (Kurt et al., 2020; Zhao et al., 2020). Adenine base editors (ABE) on the other hand, use the adenine deaminase TadA to install A-to-G edits (Gaudelli et al., 2017). Combined, these editors can restore many disease-causing mutations, in particular ABE which can repair naturally occurring C-to-T edits. Base editing takes place in a specific window, the editing window, which is usually around four nucleotides. This somewhat limits the use of base editors, since the gRNA should place the target nucleotide within this window. Besides, if multiple cytosines or adenines, for CBE/CGBE and ABE, respectively, are present in this window, all of them can theoretically be edited, resulting in bystander edits that might alter the amino acid sequence of the protein. Prediction tools have been designed, such as BE-Hive (Arbab et al., 2020), to determine which bases will be edited most efficiently. As with Cas9 nucleases, new base editors are constantly being developed with altered PAM recognition or adjusted editing windows, to increase the number of mutations that can be corrected, or improve their specificity and efficacy as well as reduce off-targets. For an overview, we refer to (Huang et al., 2021).

### Prime Editing

In 2019, another CRISPR-based tool was introduced: the prime editor (PE). Here, rather than a deaminase, a reverse transcriptase (RT) is fused to Cas9n (H840A) (Anzalone et al., 2019). In combination with a prime editing gRNA (pegRNA) that contains not just a spacer and scaffold but also a primer binding site (PBS) and an RT template, it allows to re-write all base conversions and also small insertions or deletions. Following a nick at the targeted region, the PBS anneals and the RT template is reverse transcribed. This results in an intermediate, with a 3′ and a 5′ ssDNA flap, where the former contains the edit. If this edited flap is ligated, it will create a heterodimer that can subsequently be repaired to allow incorporation of the edit. Optimizing the PE (PE2) and adding a gRNA to nick the non-edited strand (PE3) improved the efficiency of PE (Anzalone et al., 2019). In general, pegRNA design is more complicated than gRNA design for Cas9 nucleases and base editors, since there is no consensus yet on the optimal lengths of both PBS and RT. Tools have been developed to assist in the design of pegRNAs (Kim et al., 2020) but currently, multiple guides should still be empirically tested for each target.

## Cell and Animal Models of Disease

Before we discuss in detail the published proof-of-concept studies showing the feasibility to correct a variety of CFTR mutations, we first will discuss the available cell and animal models of CF, in which gene editing and a phenotypic rescue can be assessed. Cell and animal models have been invaluable for unraveling disease mechanisms in CF. They have allowed to gain insights into CF pathophysiology both at the cellular and the organ level, and have aided in the development of novel therapies to alleviate symptoms or treat the mutational defect on the DNA, RNA or protein level. For an overview of the use of different models for tailoring CFTR modulators to specific mutations, we refer to (Clancy et al., 2019). In the next section, we will discuss the different types of CF models and focus on the potential of both cell and animal models to assess a genetic correction by CRISPR-Cas or other DNA-directed editing therapies.

### Cell Models

**FIGURE 1:**
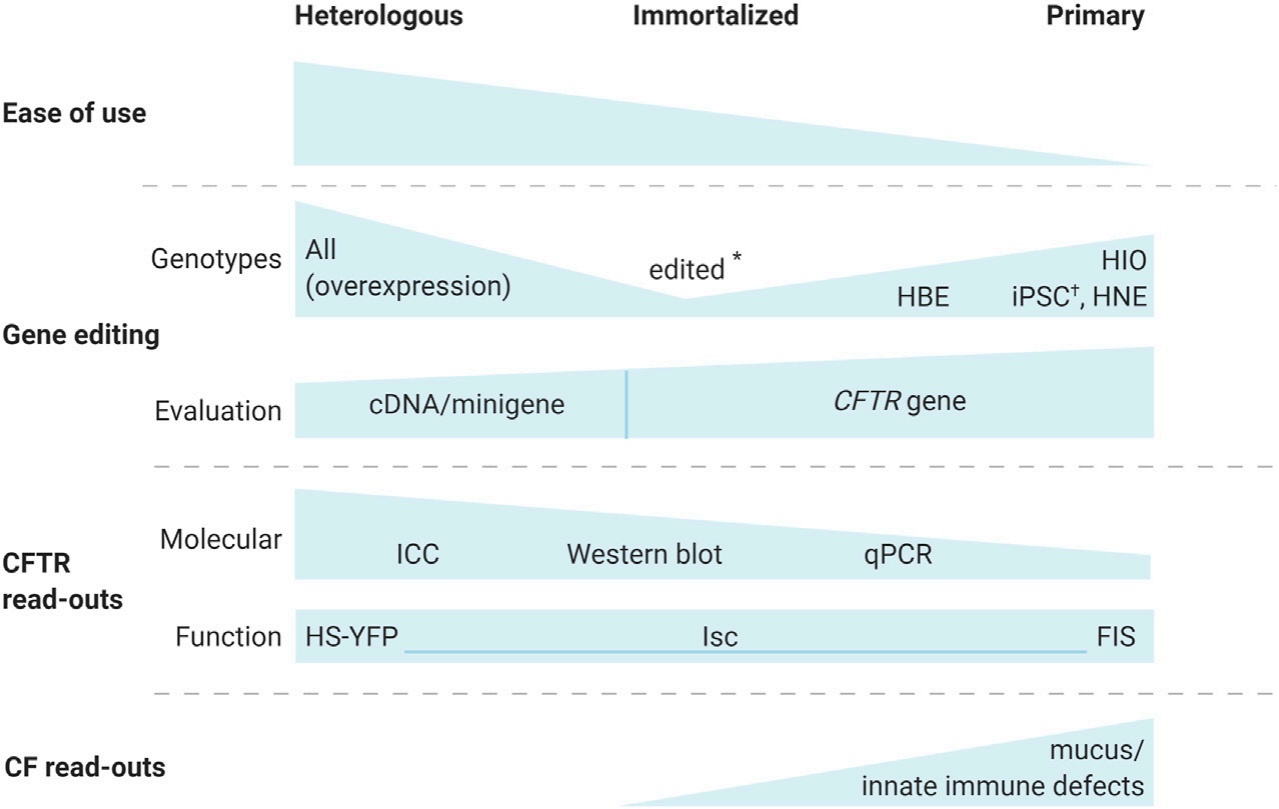
Cystic fibrosis cell models for the evaluation of gene editing strategies. Different cell-based models are available to evaluate gene editing for CF. While heterologous, CFTR overexpressing models allow the study of any CFTR variant, and are easiest to work with, their read-outs are limited to molecular and functional analysis of CFTR and genetic correction at the cDNA or minigene level. Immortalized cells, expressing the endogenous CFTR, allow correction of the *CFTR* gene, but are only available for a limited number of the more common *CFTR* genotypes. Gene editing however, has allowed to generate immortalized lines for additional genotypes (*) (Valley et al., 2019). Primary cell models are the most relevant, since they can be used for CF specific read-outs that are important in the CF phenotype, including mucus and immune defects. Traditional molecular CFTR endpoints remain challenging for primary cells. Depending on the origin of the primary cells, genotypes might be exceedingly hard to acquire i.e., human bronchial epithelial (HBE) cells from explant lungs. On the other hand, human intestinal organoids (HIO) and human nasal epithelial (HNE) cells are more readily obtained via minimally invasive procedures. Alternatively, iPSCs were edited (†) to generate isogenic lines with different genotypes (Ruan et al., 2019). A combination of different models allows the most complete cell-based evaluation of novel gene editing strategies. Abbreviations: FIS, forskolin induced swelling; HBE, human bronchial epithelial; HIO, human intestinal organoids; HNE, human nasal epithelial; ICC, immunocytochemistry; iPSCs, induced pluripotent stem cells; Isc, short circuit currents; HS-YFP, halide sensitive yellow fluorescent protein.

Different sorts of cell models have been used extensively to study CF(TR). Each cell model has its own advantages and limitations, balancing the ease of use with the possibility to employ specific read-outs and the translatability of the data gained (Figure 1). Usually, combining different complementary models allows gathering in the most complete manner all information needed to reliably confirm identified disease mechanisms and new therapeutic strategies.

#### CFTR Overexpressing Cell Models

In heterologous cell models a *CFTR* cDNA or minigene is overexpressed under an exogenous promoter. For this purpose, immortalized laboratory cell lines of human e.g., Human Embryonic Kidney (HEK)293T cells, or non-human e.g., Fischer Rat Thyroid (FRT) cells, origin are often used. The endogenous CFTR is usually not expressed in these models and thus allows functionally studying CFTR mutants, in the absence of endogenously expressed WT-CFTR. Some cell lines, such as FRT cells, can be grown into a polarized epithelium which allows electrophysiological read-outs like short-circuit current (I_sc_) measurements (Sheppard et al., 1994). Non-polarized cell lines can still be used to study CFTR function, albeit indirectly, by halide-sensitive yellow fluorescent protein (HS-YFP) quenching among others (Galietta et al., 2001). Protein maturation and subcellular localization can be evaluated using biochemical assays such as Western blotting and other antibody-based methods (e.g., ELISA, immunocytochemistry, flow cytometry) (Okiyoneda et al., 2013; Botelho et al., 2015; Ensinck et al., 2020).

The parental cell line however, is not the only consideration when choosing a heterologous cell model. The timing of expression and form in which CFTR is expressed (cDNA or minigene) are equally important. Overexpression can be either transient e.g., by plasmid transfection or electroporation, or stable, via integration into the genome after retroviral transduction or stable transfection. While transient overexpression is the quickest and most flexible method, obtained expression levels are often variable. Stable cell lines on the other hand allow to study CFTR in a more standardized manner, and have been extensively used for high throughput screening (Van Goor et al., 2006; Pedemonte et al., 2011; Liang et al., 2017) and characterization of molecular defects of *CFTR* mutations and responses to compounds (Sosnay et al., 2013; Van Goor et al., 2014; Ensinck et al., 2020) (reviewed in (Veit et al., 2016)). Results in cell lines are indicative of treatment responses in PwCF, and have been used by the FDA to evaluate repurposing of existing CFTR modulators to rare mutations. Label extensions were approved for Kalydeco™ (2017/2020), as well as Symdeko™ (2020) and Trikafta™ (2020) based on a functional rescue, measured by I_sc_, to at least 10% of WT current in FRT cell models (FDA, 2017; Vertex, 2020). These FRT cells express different *CFTR* mutations integrated at the same site using the Flp recombinase system to allow targeted cassette exchange at Flp recombination target sites (Flp-In™, Invitrogen) (Van Goor et al., 2014). In this way, isogenic CFTR-variant cell lines of choice can be rapidly generated, all with an identical genetic background. While recombinases are commonly used to generate heterologous cell models, Cas9 in principle also supports cassette exchange although with slightly altered characteristics, such as a higher risk of knock-out alleles, or insertion of extra pieces of DNA alongside the integrated transgene (Phan et al., 2017).

The *CFTR* cDNA is mostly chosen for overexpression since the *CFTR* gene spans at least 180 kb, although the exact size of *CFTR*’*s* regulatory elements is not strictly defined. In any case, it is too large to be introduced efficiently into cells, unless using artificial chromosome delivery strategies (Auriche et al., 2002). The *CFTR* cDNA (4.4 kb) contains the entire *CFTR* coding sequence but is still small enough to easily transfer into most cell types, even when an external promoter and possibly a protein tag or antibiotic selection marker are added to the construct. Protein tags like small epitopes e.g., FLAG or hemagglutinin (HA), and fluorescent proteins, *e.g.* green fluorescent protein (GFP), facilitate the detection of CFTR (Ensinck et al., 2020), as CFTR antibodies often have low sensitivity or specificity (Mendes et al., 2004). While *CFTR* cDNA allows to study the biogenesis and function of the CFTR protein, there are aspects, like splicing and nonsense-mediated mRNA decay (NMD) for nonsense mutations (also see the section *Nonsense Mutations*), which are not recapitulated in these models. Minigenes are (partial) *CFTR* cDNAs in which at least one intron is included, and used to investigate splicing mutations as well as nonsense mutations sensitive to NMD (Sharma et al., 2014; Sharma et al., 2018). It can however be difficult to identify all mutations with splicing defects based on the DNA sequence alone. For example, the *CFTR* mutation G970R (c.2908G>C) was considered a stereotypical class III gating mutant based on its normal CFTR biogenesis and response to the potentiator Ivacaftor in cDNA overexpression models (Caputo et al., 2009; Yu et al., 2012). After treatment responses to Kalydeco™ in people with this G970R mutation were lower than expected, G970R was re-investigated, this time in a primary cell model, which revealed a predominant splicing defect that resulted in exon skipping or the retention of an intron (Fidler et al., 2020). This underscores that while heterologous cell models allow to elucidate many of the molecular aspects of CFTR mutants, they should still be complemented with other, preferably primary cell models that naturally express the *CFTR* gene.

For the evaluation of gene editing in heterologous cell models, the same principle holds true. These models can be used as a first proof-of-concept, to validate guide design for example or test multiple guides or editing strategies in an easy-to-use model. Read-outs will mainly include detection of editing on the cDNA or minigene level, when allele-specific (i.e., mutation is part of the target recognition sequence, such as the protospacer) or footprint-free (i.e., *CFTR* gene is already WT and thus ‘correction’ cannot be evaluated) editing strategies are used. Targeted integration of super-exons (also see the section *Correcting Multiple Mutations at Once*) for example could be investigated on the genomic level, although the lack of endogenous CFTR expression in these models precludes any further analysis on the RNA or protein level. If a protospacer spans an exon-boundary, gRNAs should be tested on a minigene rather than on the cDNA so that gRNA design resembles the genomic sequence. Also, when splicing mutations are edited, minigene systems are particularly interesting as they allow to not only evaluate editing of the minigene, but importantly also restoration of mRNA splicing (Sanz et al., 2017; Maule et al., 2019). Alternatively, the use of cDNA models allows to characterize the CFTR biogenesis and function of edited cells in heterologous cell models not naturally expressing CFTR.

#### Immortalized Models Expressing CFTR

Cells that endogenously express CFTR provide a good complement to heterologous cell models. They are more relevant as the *CFTR* gene is expressed at its normal level and with its usual regulation. Besides, these cells usually retain more epithelial features, such as their ability to form polarized epithelia that can be grown as ALI cultures, and are of human origin (in contrast to, for example, FRT cells). The endogenous CFTR expression, for example in the cystic fibrosis bronchial epithelium cell line CFBE41o- (F508del/F508del), allows to correlate genomic *CFTR* correction with functional rescue at the protein level. One major disadvantage however is the lack of models for most of the >2100 *CFTR* mutations. To overcome this issue, several of the more common *CFTR* mutations have been introduced in the non-CF 16HBE14o-parental cell line (see the section *Introducing Mutations into CFTR*) (Valley et al., 2019). Eight genotypes are currently available, all within the same isogenic background through the CFF (CFF, 2020). To date, one of them i.e., the W1282X (c.3846G>A) human bronchial epithelium cell line 16HBE14o-, has been used to study a therapeutic rescue using ABE (also see the sections *Nonsense Mutations* and *Introducing Mutations into CFTR*) (Jiang et al., 2020).

#### Primary Cell Models of CF

Cell models directly derived from CF patients remain the golden standard for pre-clinical testing of new therapies for CF. Since CF is a multi-organ disorder which does not exclusively affect epithelia but also for example innate immune and endothelial cells (reviewed in (Declercq et al., 2019; Lara-Reyna et al., 2020), respectively), cells from many different organs can be used to understand the diverse CFTR-associated phenotypes and study a correction by rescue strategies. Human intestinal organoids, for example, are three-dimensional stem-cell based structures derived from the crypts of rectal biopsies (Sato et al., 2009). While this is an intestinal rather than an airway-based model, it is frequently used in the study of CF therapeutics, since the stimulation of these organoids with cAMP-agonist forskolin results in CFTR-dependent organoid swelling (Dekkers et al., 2013). This correlates well with improvements in PwCF in clinical trials (Ramalho et al., 2020), and this model is therefore often used for the evaluation of new small molecules and proof-of-concept gene-editing (Dekkers et al., 2016; Maule et al., 2019; Geurts et al., 2020a; Geurts et al., 2020b). HBE cultures are a logical and frequently used primary cell model in CF as lung disease is associated with most morbidity and mortality in CF patients. HBE cells are usually isolated from explant lungs, thus limiting the availability of genotypes with many of the rare mutations. When fully differentiated, these HBE cells form pseudostratified epithelia resembling the *in vivo* epithelium. Importantly they also contain the recently identified rare but high CFTR expressing ionocyte (Montoro et al., 2018; Plasschaert et al., 2018), a cell type therefore suggested important for gene targeting. While I_sc_ measurements are classically used to assess CFTR’s function as an anion channel by measuring Cl^−^ transport, investigating HCO3^-^ transport is becoming increasingly important, due to the demonstrated linear relationship between CFTR levels, HCO3^-^ transport and host defense properties (Shah et al., 2016a). Indeed, while a plateau of Cl^−^ transport is reached when 10–50% of cells express CFTR (Dannhoffer et al., 2009), explained by a limitation in Cl^−^ entry into cells at the basolateral membrane, a 50:50 mix of CF and non-CF porcine epithelial cells transported HCO3^−^ at half the rate of non-CF epithelia (Shah et al., 2016a). These findings suggest that indeed, overexpression in a smaller fraction of cells, might still result in a clinical benefit due to the strong link between HCO3^−^ transport and the host’s ability to fight bacterial infections. Host innate defense mechanisms of the airway epithelium can be measured *in vivo* or *ex vivo*, using a variety of assays, ranging from ASL pH and height, to mucociliary clearance, mucus release from the submucosal glands (SMG) and bacterial killing (for an overview, we refer to (Carlon et al., 2017)).

While the airway epithelium is the first line of defense against invading pathogens and other inhaled particulates, these cells orchestrate the further regulation of both innate and adaptive immune responses to these challenges (reviewed in (Cohen and Prince, 2012)). CF airway epithelial cells (AEC) show intrinsic immune defects, such as aberrant Toll-like Receptor trafficking, increased nuclear factor-κB (NF-κB) signaling and increased pro-inflammatory cytokine transcription (Bruscia et al., 2011; Lara-Reyna et al., 2020). A longstanding question has been whether impaired innate immune responses of immune and endothelial cells similarly, are directly linked to CFTR impairment, or rather secondary induced. On the one hand neutrophils are recruited to inflamed CF airways, even in the absence of infection, due to continuous NF-κB signaling and pro-inflammatory cytokine secretion, however, neutrophils also show a primary defect linked to CFTR dysfunction. Indeed, CFTR plays an important role in their phagocytic capacity by moving Cl^−^ ions into the phagolysosome to produce the bactericidal substance, hypochlorous acid (HOCl), a process which is impaired in CF (Zhou et al., 2013). Also, for monocytes and activated macrophages, exaggerated inflammatory responses (Meyer et al., 2009) and decreased phagocytic capacity have been reported in CF, although whether these dysfunctions are intrinsic or extrinsic, requires further investigation. In that context, CFTR modulator treatment has been reported to improve the ability of macrophages to sequester iron, which is critical to prevent both oxidative tissue damage and bacterial growth (Hazlett et al., 2020). Likewise, their ability to phagocytose and kill pathogens like *Pseudomonas eruginosa* was enhanced by modulator therapy (Barnaby et al., 2018). Both studies thus provide further support for an intrinsic CFTR defect in CF macrophages. In line with these findings, a similar pro-inflammatory phenotype was identified in CFTR-deficient endothelial cells using a transcriptomic approach, which was partially reversed by CFTR modulators (Declercq et al., 2020). Altogether, it is clear that multiple cell types contribute to the hyper-inflammatory phenotype observed throughout multiple organs in CF, requiring a broad therapeutic approach that allows correcting the multiple innate immune defects associated with dysfunctional CFTR.

Besides the eminent progressive lung disease PwCF suffer from, pancreatic insufficiency (PI) affects about 85% of the CF population. Most of them are PI soon after birth, with evidence for even structural abnormalities *in utero* (reviewed in (Singh and Schwarzenberg, 2017)). PI is caused by damage and obstruction of the pancreatic ducts. Normally, CFTR is highly expressed in pancreatic ductal epithelial cells (PDECs) and allows anions and fluid to enter the ductal lumen. This ensures that the digestive enzymes produced by the acinar cells remain in a soluble state, which is not the case in severe forms of CF (reviewed in (Wilschanski and Novak, 2013)). Besides exocrine PI, also the endocrine pancreas is frequently affected, leading to CF-related diabetes (CFRD). Whether a lack of CFTR function in PwCF is directly linked with CFRD remains unclear. In that context, Mun and colleagues have developed a patient-derived pancreas-on-a-chip to allow studying in a co-culture set-up if impaired cell-cell signaling between PDECs and islet cells leads to CFRD (Shik Mun et al., 2019). Their study indeed showed that insulin secretion was strongly decreased by inhibition of CFTR function in PDECs. This organ-on-chip system will thus allow broadening the concept of personalized medicine for CF from the well-established primary lung and intestinal cultures to a pancreatic model for studying epithelial and/or endocrine dysfunctions. Even more organ-specific cell models are emerging, such as extrahepatic and intrahepatic cholangiocyte organoids (Verstegen et al., 2020), which collectively will allow to better understand the pathological mechanisms and study a therapeutic rescue in the many organs affected in CF.

In conclusion, proof-of-concept gene editing studies have mainly focused on correcting the ion channel defect of CFTR in primary cell cultures to date (see the section *Gene Editing to Correct Mutations in CFTR*). This section however points out the importance of evaluating a therapeutic rescue beyond electrophysiological end-points as ideally all pleiotropic defects caused by CFTR dysfunction should be corrected, in order to substantially improve the multi-organ nature of CF.

### CF Animal Models: Possibilities for Evaluating Genetic Rescue Strategies

Even though cell models are increasing in complexity to better mimic the *in vivo* organ structure and function, none of them provide the same level of complexity as present in animal models of disease. Therefore, despite limitations in the translatability to humans, animal models still are of great value to study genotype-phenotype relationships and determine bioavailability, pharmacokinetics and drug efficacy in the different organs affected. In the next section, we will review the different animal models generated, from Cftr knock-out (KO) to knock-in (KI) of human CFTR mutations to humanized animal models of CF. For each of them, we will discuss their value and limitations in the context of studying a genetic correction of the gene defect in CF by gene editing technology.

#### Cftr Knock-Out Animal Models of CF

Since the identification of the *CFTR* gene, numerous animal models have been developed and widely reviewed (Grubb and Boucher, 1999; Wilke et al., 2011; Lavelle et al., 2016; Rosen et al., 2018; Semaniakou et al., 2018; McCarron et al., 2021). The conservation of this gene in mammals has made it possible to generate KO models from many different species, including mouse (Snouwaert et al., 1992; Colledge et al., 1995; Hasty et al., 1995; Rozmahel et al., 1996; van Heeckeren et al., 2004), rat (Tuggle et al., 2014; Dreano et al., 2019), rabbit (Xu et al., 2021), ferret (Sun et al., 2008; Sun et al., 2010), sheep (Fan et al., 2018) and pig (Meyerholz et al., 2010). These different models show large species-specific, and in the case of rodents, even strain-specific, phenotypic variability. For example, the KO pig and ferret present with very severe digestive and respiratory symptoms, requiring intensive postnatal symptomatic treatment such as ileostomy and antibiotics and therefore do not fully recapitulate disease progression in humans (reviewed in (Rogers et al., 2008; Sun et al., 2010)). However, these models have been extremely valuable to unravel the origin of CF lung disease (Stoltz et al., 2010; Sun et al., 2014; Keiser et al., 2015; Stoltz et al., 2015). Impaired bicarbonate secretion and reduced airway surface liquid (ASL) pH were shown to lie at the basis of viscous mucus secretions, mucostasis, impaired innate immune responses and thus the inability to efficiently clear pathogens (Stoltz et al., 2010; Hoegger et al., 2014). Mice and rats on the other hand mainly suffer from severe gastrointestinal obstruction, which requires an early and continued treatment with laxatives (Clarke et al., 1996). They show little or no respiratory phenotype, although the impairment of CFTR channel activity, measured by nasal potential difference (NPD) (Grubb and Boucher, 1999; Lubamba et al., 2008; Saussereau et al., 2013), confirms the loss of *Cftr* function. NPD is a non-invasive, translational read-out also applied in humans (Leal et al., 2008; Vermeulen et al., 2015), which allows assessing a functional rescue by gene therapy. This read-out has been used to assess the efficacy of many different gene addition rescue strategies, tested both in CF animal models (McNeer et al., 2015; Vidovic et al., 2016) and in clinical trials (Hay et al., 1995; Alton et al., 1999). For a complete overview of all *Cftr* KO animal models and their multi-organ phenotypes, we refer to the following reviews (Wilke et al., 2011; McCarron et al., 2018; Rosen et al., 2018; Semaniakou et al., 2018).

##### Species-Specific Differences Influence CF Lung Disease Phenotype

Several reasons have been put forward to explain the difference in lung disease severity between rodents and the larger CF animal models, such as the ferret and pig. A first reason relates to anatomical differences in SMG distribution throughout the proximal airways, which in rodents is restricted to the nose and the most proximal part of the trachea. CF airway pathology however has been shown to be largely attributed to defects in the SMGs as CFTR dysfunction is associated with abnormal mucus secretion and defective innate immunity (Stoltz et al., 2010; Hoegger et al., 2014; Keiser et al., 2015). It is also in that light that SMGs are proposed as one of the targets for gene therapy. Second, compensatory ion channels have been shown to alleviate the CFTR defect in CF mice. Besides Ca^2+^-activated chloride channels which provide another source of Cl^−^ secretion, ATP12A, the non-gastric form of the H^+^/K^+^-ATPase, is hardly expressed in mice, explaining the absence of ASL acidification (Shah et al., 2016b). By contrast, in CF humans and CF pigs, ATP12A leads to unchecked acidification of the ASL (Shah et al., 2016b; Scudieri et al., 2018), which is not neutralized by CFTR-mediated bicarbonate transport in the case of CF, leading to prominent lung disease. A third concept relates to differences in CFTR homology and the effect of revertant or suppressive mutations contributing to a milder lung phenotype in CF mice. For example, mouse and rat CFTR show only 78 and 76% amino acid sequence homology, respectively, compared to humans (Semaniakou et al., 2018). This difference is postulated to explain why the murine CFTR channel exhibits some alterations in pharmacological and gating properties to human CFTR (Bose et al., 2019). Among these sequence differences, suppressive or reverting variants have been identified that attenuate or reverse the severity of a pre-existing disease-causing *CFTR* mutation (Hoelen et al., 2010; Dong et al., 2012; Xu et al., 2014). In the nucleotide binding domain 1 (NBD1) sequence, the amino acid at position 539 differs between species with an isoleucine in humans, ferrets and pigs vs. a threonine in mice. The molecular consequence of this amino acid substitution was investigated in heterologous cell models expressing a chimeric hCFTR-F508del in which the human NBD1 was substituted by murine NBD1 (Dong et al., 2012), showing that the F508del protein maturation defect was attenuated in the chimeric construct. This observation supports the hypothesis that co-expression of isoleucine at position 539 and the F508del mutation presents a possible strategy to develop novel ‘humanized’ CF mouse models with more prominent lung disease, a topic that will be discussed in the section *Humanized CFTR Animal Models of CF*.

##### Value of *Cftr* KO Animal Models to Validate Gene Therapy Efficacy

Besides the possibility to correct molecular and functional end-points related to CFTR activity, these KO models and in particular those with a prominent lung phenotype, allow studying a possible improvement of human-relevant lung disease. Two different gene addition rescue strategies conducted in the newborn gut-corrected *Cftr* KO pig, reported on the reversal of impaired bacterial killing and reduced ASL pH, two important hallmarks of CF lung disease (Cooney et al., 2016; Steines et al., 2016). These studies provide the first and exciting evidence that gene therapy holds promise to improve lung disease in a large animal model of CF, with human-relevant lung disease. Extending the use of *Cftr* KO animal models to correct the endogenous *CFTR* locus by gene editing is rather limited in that sense that most gene editing strategies are designed to correct a human disease-causing mutation. The KO models thus can only be of value for gene editing strategies aiming to integrate a super-exon (Bednarski et al., 2016; Suzuki et al., 2020) in the *Cftr* locus (also see the section *Correcting Multiple Mutations at Once*), that in case of a therapeutic super-exon encompasses all the necessary exons to correct the specific *Cftr* KO present in the animal model (Wilke et al., 2011; McCarron et al., 2018; Rosen et al., 2018; Semaniakou et al., 2018).

#### Human Relevant *CFTR* Mutations in the Animal Orthologue: Knock-in CF Animal Models

The development of genetic engineering tools has made it possible to generate different models expressing a human relevant *CFTR* mutation. Several frequent disease-causing *CFTR* mutations have been inserted into the animal *Cftr* gene to generate knock-in models (Table 1). Of the currently 2100 *CFTR* mutations reported, only five have been used to date to develop CF animal models, including R117H (c.350G>A), G480C (c.1438G>T), F508del, G542X (c.1624G>T), and G551D (c.1652G>A). Overall, these models have a generally less severe phenotype than that observed in humans, likely due to the above discussed reasons, and also less severe than their respective KO counterparts per species. As with the KO models, a difference in phenotype is observed according to species (Table 1).

**TABLE 1:** Knock-in CF animal models.

Species	Mutation	Editing technology	Insertion	CF phenotype	References
Mouse	F508del	Plasmid	HR	Peritonitis, intestinal obstruction	Colledge et al. (1995)
				Electrophysiological abnormalities in trachea and colon epithelium	
				Distention and mucus hyper-accumulation in intestinal glands and colon mRNA expression lower than WT	
Mouse	F508del	Plasmid	HR	Growth retardation	van Doorninck et al. (1995)
				Hypertrophy of goblet cells in intestine	
				Residual CFTR function in nasal, intestinal and gallbladder epithelium	
Mouse	F508del	Plasmid	HR	Growth retardation	Zeiher et al. (1995)
				Peritonitis, intestinal obstruction
				Electrophysiological abnormalities in nasal, intestinal and pancreatic epithelium
				Inflammatory cells in broncho-alveolar lavage
Mouse	G551D	Plasmid	HR	Intestinal obstruction, peritonitis	Delaney et al. (1996)
				Electrophysiological abnormalities in nasal, tracheal and intestinal epithelium	
Mouse	G480C	Plasmid	HR	Hypertrophy of goblet cells in intestine	Dickinson et al. (2002)
				Electrophysiological abnormalities in nasal epithelium	
Mouse	R117H	NR	HR	Growth retardation	van Heeckeren et al. (2004)
				Electrophysiological abnormalities in nasal epithelium	
Pig	F508del	rAAV	HR	Intestinal obstruction	Rogers et al. (2008), Ostedgaard et al. (2011)
				Liver steatosis	
				Pancreatic abnormalities	
				CF lung disease: Airway obstruction, host-defense defect	
				Residual CFTR function in nasal and pancreatic epithelium	
Mouse	G542X	CRISPR/Cas9	HR	Growth retardation	McHugh et al. (2018)
				Intestinal obstruction	
				No CFTR activity in airway and intestinal epithelium	
Rat	F508del	CRISPR/Cas9	HR	Growth retardation	Dreano et al. (2019)
				Abnormal dentition	
				Intestinal obstruction	
				Residual CFTR function in airway and colon epithelium	
Ferret	G551D	rAAV	HR	Intestinal obstruction mRNA expression reduced in lung and intestine	Sun et al. (2019)
				Protein expression reduced in lung	
				Electrophysiological abnormalities in intestinal organoids, jejunum and pancreatic ductal epithelium	
				Airway obstruction	
				Reproductive tract malformations	
Rat	F508del	CRISPR/Cas9	HR	Growth retardation	McCarron et al. (2020)
				Abnormal dentition	
				Intestinal obstruction	
				Residual CFTR function in nasal epithelium	
				Reproductive tract malformations	
Rat	G542X	CRISPR/Cas9	HR	Growth retardation	Sharma et al. (2020)
				Abnormal dentition	
				Intestinal obstruction	
				No CFTR activity in airway and intestinal epithelium mRNA degradation (NMD)	

Abbreviations: CRISPR/Cas, Clustered Regularly Interspaced Short Palindromic Repeats/CRISPR associated protein; HR, Homologous recombination based on the presence of flanking homology arms; KO, Knock-out; NMD, Nonsense-mediated decay; NPD, Nasal potential difference; WT, Wild-type.

Regarding the F508del mutation, rodent models show little airway pathology, but rather a predominant intestinal obstruction, which can be improved by a laxative diet, in contrast to the F508del pig. Although the F508del pig shows very severe digestive and lung pathology, the poor survival of this model makes longitudinal studies challenging (Rogers et al., 2008). Similarly to F508del, the G480C mutation shows impaired processing and trafficking (Smit et al., 1995; Ferec and Cutting, 2012) and likewise is coupled to a severe phenotype in patients (Kristidis et al., 1992). Contrary to human disease, the G480C mouse model only showed hypertrophy of goblet cells in the intestine and an organ-specific electrophysiological defect (defective Cl^−^ transport in the nose but not in the cecum) (Dickinson et al., 2002). These species-specific phenotypic differences can likely be attributed to the differences in anatomy, ion channel composition and CFTR protein sequence, as discussed above. A complete overview of all knock-in animal models of CF and their organ-specific phenotypes is given in Table 1. Despite these species-specific variations, these models remain of interest for the preclinical evaluation of new therapies, as well as for unraveling the mechanisms of disease initiation and progression, while considering the limitations and differences with respect to their translation to humans (Shah et al., 2016b; Scudieri et al., 2018). First, a major asset of these different animal models is that they recapitulate the complex multi-organ disease seen in PwCF and thus allow to evaluate treatment efficacy in different organs (Sun et al., 2019). Second, the complex pulmonary environment of PwCF represents a major extracellular barrier to drug and gene therapy delivery which cannot be fully recapitulated in *in vitro* models, underscoring the need to study drug penetration through the multiple barriers in relevant animals with severe lung disease (Stoltz et al., 2010; Hoegger et al., 2014; Sun et al., 2014; Keiser et al., 2015). Finally, they allow to address long-standing questions which remained unanswered even in primary cell models, such as the presumed irreversibility of exocrine pancreas insufficiency and the obliteration of the vas deferens, both already present prenatally. The first *in utero* administration of potentiator Ivacaftor (VX-770) in the G551D ferret allowed to improve pancreatic exocrine function, as well as rescue vas deferens and epididymis development, suggesting an important role for CFTR early in life in establishing organ function (Sun et al., 2019).

##### Value of Animal Models with Knock-in of Human *CFTR* Mutations to Validate Gene Therapy Efficacy

The availability of human relevant *CFTR* mutations in animal models is advantageous for evaluating mutation-specific treatments such as gene editing aiming at an *in vivo* delivery approach, at least for those mutations currently modeled in animals (Table 1). In that regard, successful *in vivo* gene editing of the F508del mutation in mice has been demonstrated using triplex-forming peptide nucleic acids (PNA) (also see the section *F508del*) (McNeer et al., 2015). By NPD, a partial but significant recovery in CFTR function was demonstrated in the nose. In the lung, a reduction in the number of inflammatory cells in broncho-alveolar lavage fluid (BALF) was indicative of a rescue by F508del-specific PNA treatment. This study hence presents promising work on the first steps toward an *in vivo* gene editing therapy, here tailored to the most common F508del mutation, further showing which read-outs can be used in a knock-in mouse model without prominent lung pathology.

In general, the severe morbidity and mortality associated with CF lung disease, have guided gene therapy efforts toward a lung-directed approach since decades (reviewed in (Carlon et al., 2017; Sondhi et al., 2017)). In that regard, animal models that recapitulate more prominent lung disease and the thereby associated extracellular barriers hampering efficient gene transfer, such as viscous mucus, chronic inflammation and infection, underscore the importance of including these models in the therapeutic development pipeline (Cooney et al., 2016; Steines et al., 2016) (and reviewed in (Mottais et al., 2017). A limitation of current knock-in models, is that despite the *CFTR* gene being phylogenetically conserved between mammals, sequence variations require a species-dependent gRNA, ZFN or TALEN design. Indeed, McNeer and colleagues had to construct two distinct PNA and donor templates to correct either the human F508del in primary HBEs or the murine F508del in the CF mouse model used (McNeer et al., 2015). While at first sight, this species-specific testing might limit the direct translation of editing tools to humans, other factors likely are of more importance, such as the delivery vehicle which determines tropism and targeting efficiency. Nevertheless, one of the strategies to overcome cross-species genotype and phenotype variations, is to develop humanized animal models of CF.

#### Humanized *CFTR* Animal Models of CF

Humanized *CFTR* animal models of CF can be divided in two main classes (Table 2). The first class includes transgenic models with a random insertion of the human *CFTR* cDNA or full gene into the genome of *Cftr* KO mice. The second class corresponds to humanized models in which the *Cftr* mouse gene is replaced by the human *CFTR* cDNA via homologous recombination strategies. A first transgenic mouse model was developed in 1994, the so-called ‘gut rescued’ KO mouse, where the human *CFTR* cDNA under control of the intestinal fatty acid binding protein (FABP) promoter was randomly inserted into a *Cftr* KO model (Zhou et al., 1994). The local expression of this transgene made it possible to rescue CF intestinal pathology and thus increase the survival of these transgenic mice, thereby increasing the overall applicability to study therapeutic interventions among others. In a similar manner, a gut-rescued KO ferret and pig were made, though with the animal orthologue (Sun et al., 2010; Stoltz et al., 2013). Also, human relevant *CFTR* mutations have been introduced into humanized CF animal models, a first one being the most prevalent nonsense mutation, G542X. Similar to the gut corrected models, this transgenic mouse was constructed from a *Cftr* KO model in which the *G542X-CFTR* cDNA under the control of the FABP promoter ensured local expression of G542X-CFTR in the gastro-intestinal tract. So far, this model has been used to test translational read-through inducing drugs in their ability to pharmacologically correct premature termination codons (PTCs) in CFTR (Du et al., 2002; Du et al., 2006). These corrections were measured by analysis of survival rate, CFTR function (I_sc_) and hCFTR expression in the intestine.

**TABLE 2:** Introduction of human *CFTR* to generate humanized CFTR animal models.

Species	Background	Mutation	Construction	Insertion	CF phenotype	References
Mouse	*Cftr* ^tmUnc^ null mice	h*CFTR* cDNA under control of FABP promoter	Plasmid	Random insertion	Gastro-intestinal pathology rescued by gut-specific expression of *hCFTR* under the FABP promoter	Zhou et al. (1994)
Mouse	*Cftr* ^tm1Cam^ null mice	h*CFTR* full gene + regulatory elements (70 kb of flanking sequence)	YAC	Random insertion	Different function according to the founder	Manson et al. (1997)
					CFTR function rescued in colon, jejunum and cecum with h*CFTR* expression under endogenous promoter	
Mouse	*Cftr* ^tm1Cam^ null mice	*hG542X-CFTR* cDNA under control of FAPB promoter	Plasmid	Random insertion	Growth retardation	Du et al. (2002)
					Intestinal obstruction	
					Occasional weak cAMP-stimulated current in intestinal epithelium	
Mouse	*Cftr* ^tmUnc^ null mice	*hCFTR* full gene + regulatory elements (40.1 kb	BAC	Random insertion	Abnormal dentition	Gawenis et al. (2019)
		At 5′; 25 kb at 3′ end of *hCFTR* gene)			Morphological and functional (nasal and intestinal mucosa) rescue with hCFTR expression under endogenous promoter	
Rat	WT sprague-dawley rat	*h*G551D-*CFTR* cDNA (2–27 exons) under control of endogenous promoter	ZFN and super-exon	HR	Growth retardation	Birket et al. (2020)
					Abnormal dentition	
					Intestinal obstruction	
					ASL depletion, PCL decrease, mucus transport decrease and presence of viscous mucus	

Abbreviations: ASL, airway surface liquid; BAC, Bacterial artificial chromosome; FABP, Fatty acid binding protein; hCFTR, human CFTR; HR, Homologous recombination; KO, Knock-out; PCL, periciliary liquid; YAC, yeast artificial chromosome; WT, wild-type; ZFN, Zinc finger nuclease.

Two other transgenic models have been generated using yeast artificial chromosomes (YAC) (Manson et al. (1997)) and recently also bacterial artificial chromosomes (BAC) (Gawenis et al., 2019). They have as most prominent advantage the possibility to incorporate the entire h*CFTR* gene, including all necessary regulatory elements that control its expression, which is impossible using plasmid or viral vector technology. The BAC transgenic mouse with functional hCFTR was generated with the specific purpose to enable the subsequent generation of mouse models with *hCFTR* mutations, to support future *in vivo* testing of new CF therapies, pharmacologic or gene therapeutic (Gawenis et al., 2019). Phenotypically, all CF-specific organ pathologies were rescued to WT levels, with the exception of the abnormal dentition. Follow-up studies will likely follow, reporting on the introduction of human *CFTR* mutations in this BAC transgenic mouse model, which will allow studying mutations and rescue strategies in a congenic background.

Recently, a humanized transgenic G551D rat model was generated (Birket et al., 2020), the rationale being that pharmacological responses are often species-specific, such as the differential Ivacaftor response of mouse vs. human F508del-CFTR (Bose et al., 2019). A *hCFTR* cDNA super-exon, spanning exon 2–27 was inserted using ZFNs with a 5′ insertion site into the rat *Cftr* gene just beyond intron 1. This targeted insertion allows G551D-CFTR expression from the endogenous rat promoter. The G551D rat model developed a CF phenotype similar to the KO rat and responded, as hypothesized, to potentiator Ivacaftor with a restoration of nasal and tracheal potential difference. Besides a functional recovery of CFTR, also lung disease relevant parameters were normalized, such as airway mucus viscosity, mucociliary clearance and ASL height, the latter implying a restored hydration of the airways.

##### Value of Humanized CF Animal Models to Validate Gene Therapy Efficacy

Generating humanized animal models of disease is an interesting approach that is gaining ground in biomedical research because of several advantages. First, disease phenotypes might more closely resemble human disease, compared to knock-in models where mutations are introduced into the animal orthologue, as interspecies sequence variations and reverting mutations are abolished. However, other confounding factors remain, such as differences in anatomy and ion channel composition. Second, expressing the human protein in an *in vivo* model is advantageous for studying drug responses, both small molecules and gene therapies, as pharmacodynamics and -kinetics ultimately determine the net uptake and tissue-specific responses to the drug. Third, the delivery of very large inserts by artificial chromosomes (YAC, BAC) opens up possibilities to not only insert cDNA, mini-genes or super-exons, but even complete genes with their regulatory elements that contribute to the tissue-specific and dynamic expression of hCFTR. This level of transcriptional control allows a more faithful and human-relevant evaluation of specific therapies for which the non-coding elements contribute to the rescue efficacy, such as PTC modulating agents and therapies aiming to correct splicing mutations (reviewed in (Clancy et al., 2019)).

On the other hand, humanized animal models hold possible limitations which should be considered. A potential risk is that the humanized protein e.g., CFTR, might interact differently with the animal-specific cellular environment, for example based on altered protein-protein interactions. In that light, the humanized G551D rat did not show a difference in the amiloride-sensitive transepithelial potential difference compared to WT-CFTR (Birket et al., 2020). We hypothesize that this might reflect a distinct, non-inhibiting interaction between human CFTR and the murine epithelial Na^+^ channel. Another potential limitation of humanized models is that the animal transcription factor orthologues might not bind the inserted human regulatory regions with the same specificity, potentially resulting in altered hCFTR expression. Further, other cellular mechanisms, such as RNA splicing or chaperone-assisted CFTR folding could potentially differ between species and hinder the utility of reliable humanized animal models (Zhu et al., 2019). These theoretical limitations should be kept in mind and experimentally verified during the development process of the humanized animal model to ensure their value in the therapeutic discovery pipeline.

Possible future perspectives include the development of even closer animal mimics of human CF disease such as compound heterozygous genotypes (i.e., two different mutant *CFTR* alleles), which is frequently the case in CF patients. This would allow a better understanding of the complex genotype-phenotype relations leading to organ-specific CF pathophysiology, as well as more reliable functional responses in the development of new therapeutics.

## Gene Editing to Correct Mutations in *CFTR*: What has Been Done?

Gene editing to correct mutations at the endogenous *CFTR* locus remains a rather recent field, compared to other therapeutic strategies for CF, like gene addition and CFTR modulators. Nevertheless, many proof-of-concept studies in CF cell models, including primary intestinal organoids, airway cultures and induced pluripotent stem cells (iPSCs), have already shown that correction of *CFTR* mutations is feasible. A comprehensive overview of all these studies is given in the next section.

Currently, the use of nucleases combined with HDR has been most widely investigated. Gene editing usually is highly mutation specific, and since CF is caused by at least 360 different mutations, covering base conversions as well as small and large insertions and deletions, multiple strategies will be needed to model all mutations or develop a treatment for all PwCF. For an overview of how many mutations can theoretically be covered by each gene editing strategy, we refer to (Maule et al., 2021). The *CFTR* gene spans a >180 kb region and some parts will be more suited for editing compared to others e.g., when it comes to the availability of PAM sites or GC content. Therefore, most likely different strategies will need to be tested and tailored to each specific mutation.

Before these proof-of-concept studies can be translated toward a therapy, further optimizations are needed and additional questions remain to be addressed. For example, many strategies to date used antibiotic selection to enrich edited cells, which is not compatible with *in vivo* therapies. Will genetic treatment be most effective when editing tools are delivered *in vivo,* or rather as an *ex vivo* cell therapeutic approach, requiring successful engraftment in the target organ? For some disorders this is easily decided, such as the *ex vivo* route for hematological disorders (Corbacioglu et al., 2020) and the *in vivo* strategy for inherited blindness (Editas, 2020), but for CF this question remains unanswered. Delivery of the editing machinery will have to be optimized since plasmid, mRNA or ribonucleoprotein (RNP) transfection or electroporation, while efficient in cell culture, does not translate to direct *in vivo* delivery to patients. Unlike the highly specific gene editors themselves, translation from proof-of-concept editing in cells toward a patient-directed therapy will likely be more generic, as one or a few optimized delivery vehicles can incorporate any given gene editing tool set.

Despite the current lack of immediate translational potential, these proof-of-concepts report on the efficiency and safety of editing specific *CFTR* mutations in relevant cell and animal models of CF. These preliminary but encouraging studies have set the stage to further invest in the translation of gene editing toward its future in patient use. Importantly, gene editing technology has leveraged the generation of CF cell and animal models, which allows gaining better insights into CF disease mechanisms which might be mutation specific, and hence require a tailored therapeutic intervention. In the next section we will first discuss different proof-of-concept gene editing examples for CF, correcting F508del, intronic splicing mutations, nonsense mutations, and the possibility to correct multiple mutations at once with super-exons, as well as introducing these mutations to generate CF models. Next, we will also discuss how these could be translated into therapies.

### Proof-of-Concept Gene Editing for CF

#### F508del

So far, most CF gene editing efforts have focused on restoring F508del, as this is the most common *CFTR* mutation, accounting for ∼70% of all CF alleles. Early studies used ZFNs to target the F508del region in heterologous cell lines, to validate this region could be targeted by ZFNs but did not yet correct the mutation (Maeder et al., 2008). Several years later, F508del was corrected using ZFNs and a donor DNA template containing the wild type (WT) sequence in CF tracheal epithelial cells, albeit with efficiencies below 1% (Lee et al., 2012). This could be improved to 1.9% with the use of Cas9 and an optimized repair template (Hollywood et al., 2016). Since the latter contained the complete exon 11 (containing the F508del mutation) this same strategy could potentially also be applied to any of the other twenty CF-causing mutations in this exon. Correction of F508del and I507del was also obtained using HDR following ZFN-induced DSBs in iPSCs from PwCF who were compound heterozygous for both mutations (Crane et al., 2015). Corrected cells were enriched by including a puromycin-thymidine kinase cassette in the HDR template, which allowed positive puromycin selection and subsequent negative selection with ganciclovir after excision of the selection cassette by Cre recombinase. When corrected clones were subsequently differentiated into epithelial cells, they expressed CFTR which matured correctly and functionally responded in I_sc_ measurements like WT samples. A similar selection cassette was used to correct F508del/F508del iPSCs using a Cas9 nuclease, but excision was mediated via a piggyBac transposase rather than Cre, thereby providing footprint-free editing with ∼15% efficacy (Firth et al., 2015). When differentiated into proximal airway epithelial cells these clones showed corrected CFTR maturation and function, by Western blot and I_sc_ analysis, respectively.

Alternatively, F508del-containing iPSCs were corrected using TALENs and an SDF (small/short DNA fragment) donor of around 500 nt (Sargent et al., 2014; Suzuki et al., 2016). After several rounds of selection-marker-free selection, corrected clones were differentiated into airway cells, demonstrating functional CFTR. F508del homozygous iPSCs were also corrected using Cas9 and F508del-specific sgRNAs with ∼2.4% efficacy (Smirnikhina et al., 2020). Recently, puromycin selection of TALEN-corrected iPSCs resulted in ∼10% F508del correction efficiency (Fleischer et al., 2020). Clones were differentiated into intestinal organoids and their functional rescue was shown by CFTR-dependent organoid swelling upon stimulation with the cAMP agonist forskolin. Primary intestinal organoids homozygous for the F508del mutation had previously also directly been edited using Cas9 and an HDR template with a puromycin selection cassette (Schwank et al., 2013).

All of these examples have in common however, that either F508del was gene edited at low efficiency or that one or multiple rounds of selection were required in order to enrich the edited cells. Moreover, analysis of CFTR function took place on sequencing-verified, corrected clones. Neither selection nor screening of clones is translatable into an *in vivo* gene therapy approach, so for this strategy, editing efficiencies should be further improved. By optimizing electroporation of CRISPR-Cas9 components as RNPs combined with a single strand oligodeoxynucleotide (ssODN) repair template, F508del correction efficiencies of ∼22% could be achieved without selection in iPSCs (Ruan et al., 2019). When repaired clones were differentiated into proximal lung organoids, forskolin-induced organoid swelling confirmed the genetic correction of F508del to WT or carrier (the latter phenotypically similar to WT). Also, nuclease-independent HDR-mediated editing of F508del by means of triplex-forming peptide nucleic acids (PNAs) has been described (McNeer et al., 2015). Formation of a PNA/DNA/PNA triplex will initiate DNA repair mechanisms to allow recombination. For an overview of the use of PNAs in gene editing we refer to (Ricciardi et al., 2018). CFBE41o-cells, a frequently used human F508del/F508del cell line, were corrected with an efficiency of 9.2%, while mouse *F508del-Cftr* was corrected *in vivo* at efficiencies of 5.7 and 1.2% in nasal and pulmonary epithelium, respectively, after intranasal delivery of nanoparticles (McNeer et al., 2015). The *in vivo* editing partially rescued CFTR function in the nasal epithelium, as measured by NPD.

Recently, studies have focused on correcting *F508del-CFTR* in airway progenitor cells, *i.e.* basal cells, a potentially interesting cell type for *ex vivo* stem cell therapy (Suzuki et al., 2020; Vaidyanathan et al., 2020). RNP electroporation and delivery of a donor template by adeno-associated viral vector serotype 6 (AAV6) transduction resulted in F508del correction in 28% of alleles in F508del/F508del samples and 42% in compound heterozygous samples (Vaidyanathan et al., 2020). ALI cultures derived from these upper airway basal cells (UABCs) showed rescued CFTR maturation and function. Alternatively, ZFN mRNA electroporation in combination with AAV6 donor template transduction resulted in 31% allele correction and restoration of CFTR expression and function in ALI cultures (Suzuki et al., 2020).

Due to the fact that F508del results from a three-nucleotide deletion, it cannot be corrected by base editing. However, it is an interesting target for prime editing, as this editor allows installing small insertions, like the CTT insertion needed to correct F508del. Very recently, the first prime editing attempts for the F508del mutation were reported in primary intestinal organoids, currently still a pre-print (Geurts et al., 2020b). Although efficiencies were very low compared to HDR gene editing, electroporation of PE3 (optimized prime editor, pegRNA and additional ngRNA) plasmids resulted in a genetic correction and recovery of CFTR channel function in a few corrected organoid clones.

#### Intronic Splicing Mutations

As CF is caused by loss-of-function mutations throughout the *CFTR* gene, the use of nucleases to correct disease-causing mutations without subsequent repair by HDR is restricted to a small group of specific mutations. Intronic mutations can create cryptic donor or acceptor splice sites which result in the incorporation of extra nucleotides into the *CFTR* mRNA. Often, this causes frameshifts, completely disrupting the remainder of the protein sequence. However, as low levels of transcripts get normally spliced, these intronic or cryptic splicing mutations are generally associated with relatively mild CF (as determined by pancreatic sufficiency) (CFTR2, 2021). Since intronic regions are less conserved compared to exons and do not code for proteins, removal of the cryptic splice site can be achieved by NHEJ which is more efficient than HDR and abolishes the need to deliver a donor template. Excising or disrupting the cryptic splice site by inducing indels is hence sufficient to restore the WT *CFTR* mRNA. In that regard, the feasibility of correcting the intronic splicing mutations 1811 + 1.6kbA > G (c.1680-886A > G), 3272-26A > G (c.3140-26A > G) and 3849 + 10kbC > T (c.3718–2477C > T) has been shown by excising these mutations using SpCas9 and two flanking sgRNAs in cell lines overexpressing the respective *CFTR* minigenes. This resulted in up to 90% of corrected minigene splicing depending on the mutation targeted (Sanz et al., 2017). In patient-derived airway cells and intestinal organoids 3272-26A > G and 3849 + 10kbC > T were corrected making use of a single mutation-specific sgRNA in combination with Cas12a (Maule et al., 2019). Specifically, 85% of mutant alleles were successfully edited in compound heterozygous organoids, while less than 1% of the other *CFTR* allele was targeted. CFTR activity of gene edited organoids reached similar levels as those transduced with a lentiviral vector encoding *WT-CFTR* cDNA without the need for prior enrichment, underscoring the efficiency and safety of this gene editing approach. While for CF this strategy to treat intronic splicing mutations has only been studied as a proof-of-concept so far, a similar strategy to cure Leber congenital amaurosis is currently already being investigated in a clinical setting (EDIT-101, NCT03872479 (Maeder et al., 2019)).

#### Nonsense Mutations

Nonsense mutations account for ∼5% of disease-causing alleles in CF and are associated with severe CF (CFTR2, 2021). They introduce PTCs which give rise to truncated proteins. Due to the presence of the PTC there are exon boundaries on the pre-mRNA after the stop codon, which often subjects it to NMD (reviewed in (Kurosaki and Maquat, 2016)). NMD leads to very low expression levels of mutant transcripts, further complicating rescue by small molecules. To date, no causal therapies have been approved for nonsense mutations in CF. However, drug discovery efforts are ongoing to identify more effective translational read-through inducing drugs than the most advanced drug tested so far in clinical trials i.e., Ataluren, which due to lack of efficacy in patients was discontinued (Kerem et al., 2014). In parallel, also gene editing strategies are being pursued as an alternative with the potential of even providing a cure. In that light, ABE was evaluated to correct a number of nonsense mutations (R553X (c.1657C > T), R785X (c.2353C > T), R1162X (c.3484C > T) and W1282X) in primary intestinal organoids (Geurts et al., 2020a). ABE plasmids were electroporated and treated cells were enriched via selection. This resulted in editing efficiencies between 1.43 and 8.8%, depending on the mutation. Individual corrected organoids showed CFTR dependent swelling upon forskolin stimulation, confirming their functional correction and allowing to phenotypically select corrected organoids. Interestingly in a pre-print, for the R785X mutation, correction efficiencies were compared between HDR, ABE and prime editing in primary intestinal organoids. ABE resulted in approximately 6 times more forskolin-responsive organoids than HDR and prime editing, the latter both showing similar efficiencies (Geurts et al., 2020b).

W1282X, the second most common CF-causing nonsense mutation, has also been corrected by mRNA electroporation of ABE in a W1282X-encoding cell line, resulting in ∼26% base conversion which rescued 10% of CFTR protein expression (Jiang et al., 2020). Of note, the editing window contained a second adenine, which was edited more efficiently than the target adenine (∼45%) and introduced a missense mutation (Q1281R). This bystander edit, however, did not preclude a functional rescue of CFTR activity when both edits were present on the same allele. Nevertheless, it shows the importance of functionally evaluating the effect of possible bystander edits when applying base editing. An alternative approach for W1282X that focused on rescuing its expression by preventing NMD has also been described. The simultaneous use of two gRNAs, the first targeting exon 23 in an W1282X-specific manner, and the second exon 27, induced a 24 kb deletion which removed the exon boundaries downstream from the W1282X mutation, thereby preventing NMD (Erwood et al., 2020). Indeed, this deletion resulted in a 2.4-fold increase in the expression of the truncated mRNA and protein in human bronchial epithelial cells. The truncated protein could functionally be rescued by existing CFTR correctors and potentiators, providing a novel combination treatment potentially applicable to patients harboring this C-terminal nonsense mutation. While this approach thus allows to functionally rescue W1282X, it will likely not be translatable to other, more N-terminally located nonsense mutations, such as the most common nonsense mutation G542X, because this strategy is based on downstream deletion of the remainder of the *CFTR* gene.

#### Correcting Multiple Mutations at Once: Super-Exons

Most examples that have been discussed so far have focused on highly mutation-specific strategies using nucleases, base and prime editors. There is however another strategy that uses HDR to potentially correct not one but many mutations at the same time: targeted integration of a super-exon (reviewed in (Mention et al., 2019)). Super-exons code for the part of the *CFTR* cDNA downstream of the integration site, which get integrated into the endogenous *CFTR* locus. In this way, regulation of protein expression remains unaltered which is potentially advantageous over classical gene addition where an entire *CFTR* cDNA under control of an external promoter is randomly integrated into the host genome. Super-exons allow to correct all mutations included into this partial cDNA, so ultimately the integration site will determine which mutations are rescued by a super-exon strategy. In order to rescue F508del (and all other mutations located in exons 11–27 of *CFTR*) a super-exon was integrated into exon 11 in CFBE41o-cells using ZFNs followed by HDR (Bednarski et al., 2016). The super-exon also contained a puromycin cassette which allowed selection of edited clones and resulted in ∼10% integration efficiency. In a corrected clone, CFTR activity was comparable to a non-CF sample. Recently, a similar approach was investigated by integrating a cDNA containing exons 9–27 into intron 8 or exons 8–27 into intron 7 of *CFTR*, using ZFN electroporation combined with AAV6 transduction to deliver the repair template in patient derived airway basal cells (Suzuki et al., 2020). Integration efficiencies ranged from ∼36 (in intron 7) to ∼56% (in intron 8), restoring CFTR activity to ∼30 and ∼40% of non-CF levels, respectively, in ALI.

#### Introducing Mutations into *CFTR*

Gene editing can also be applied to create new CF models by introducing disease-causing mutations into the *CFTR* gene. One such application is to generate isogenic cell lines i.e., CF and non-CF lines with the same genetic background, to allow studying the molecular defects of *CFTR* mutations in the absence of variable donor backgrounds. This is of particular interest in CF as responses to CFTR modulators have been highly variable between patients with the same *CFTR* genotype in clinical trials (Heijerman et al., 2019; Middleton et al., 2019), as well as in patient-derived samples (Dekkers et al., 2016), likely due to modifier genes and differences in the cellular environment. Isogenic cell lines have been generated starting from the often-used 16HBE14o-cell line, using CRISPR-Cas RNPs and a ssODN donor template to install the F508del, G542X, and W1282X mutations into the *CFTR* gene (Valley et al., 2019). Subsequently, the polymorphism V470M was introduced into the gene edited F508del cells, as F508del is exclusively associated with M470 which might influence disease characteristics. G542X and W1282X edited cells showed NMD levels in line with primary cell material, and responded well to NMD inhibition or PTC readthrough agents, underscoring the translational potential of these gene edited isogenic cell lines. Of note, while homozygous editing was obtained, deep sequencing of the *CFTR* gene revealed an intronic insertion from the immortalization process of the parental 16HBE14o-cells on one of the two alleles, which was consequently knocked out. Alternatively, the F508del, G551D and G542X mutations have been introduced into non-CF iPSCs, using a similar strategy, as well as the compound heterozygous F508del/G551D genotype (Ruan et al., 2019). While so far, the focus has been on generating models for the more common *CFTR* mutations, gene editing opens up opportunities to also model rare *CFTR* mutations. As homozygous samples are often not available and the presence of the other mutant *CFTR* allele (most commonly F508del) confounds the interpretation of the functional response measured, isogenic cell lines will allow to unbiasedly determine the molecular defect and treatment responses of each of these mutations.

### From Proof-of-Concept to Therapy

As proof-of-concept studies have shown the feasibility to correct CF at the genomic level, enthusiasm and expectations are booming to rapidly translate these studies into therapies. However, before gene editing can be translated toward a treatment for PwCF, several questions need to be addressed. As mentioned previously, gene editing for CF could either be *ex vivo* or *in vivo.* So far, gene editing that has made it into clinical trials mainly consists of *ex vivo* examples (reviewed in (Hampton, 2020; Hirakawa et al., 2020)). *Ex vivo* cell therapy for CF however is a recent field, and no clinical trials were performed yet, either for gene editing or classical gene addition gene therapy. After sufficiently efficient correction, it will be key to successfully engraft the edited cells into the airway epithelium. Particularly in CF, this airway is severely inflamed and covered with thick mucus, which will further complicate this process. Nevertheless, edited UABCs from an F508del/F508del donor could be grown on an FDA-approved biodegradable scaffold while maintaining their differentiation potential and CFTR expression, a first step toward translation to an *ex vivo* cell therapy (Vaidyanathan et al., 2020). For an overview of the challenges for cell-based therapies for CF, we refer to (Berical et al., 2019). For *in vivo* gene therapy on the other hand, there have been many clinical trials in the past investigating gene addition gene therapy for CF (reviewed in (Sondhi et al., 2017)), although eventually all were discontinued. From these studies however, it became evident that sufficient numbers of cells need to be targeted, preferably progenitor cells i.e., basal cells, to provide long-term *CFTR* correction rather than the transient effects that were observed in clinical trials so far. Alternatively, if long-term correction cannot be achieved, repeated administrations will be necessary to maintain a functional “cure” for CF. Immune responses, both innate and pre-existing or acquired neutralizing antibodies, against vector components (reviewed in (Shirley et al., 2020)), gene editing components (reviewed in (Mehta and Merkel, 2020)), or even the CFTR protein (Figueredo et al., 2007; Limberis et al., 2007), might hamper efficacy of gene therapies and should be taken into consideration.

**FIGURE 2:**
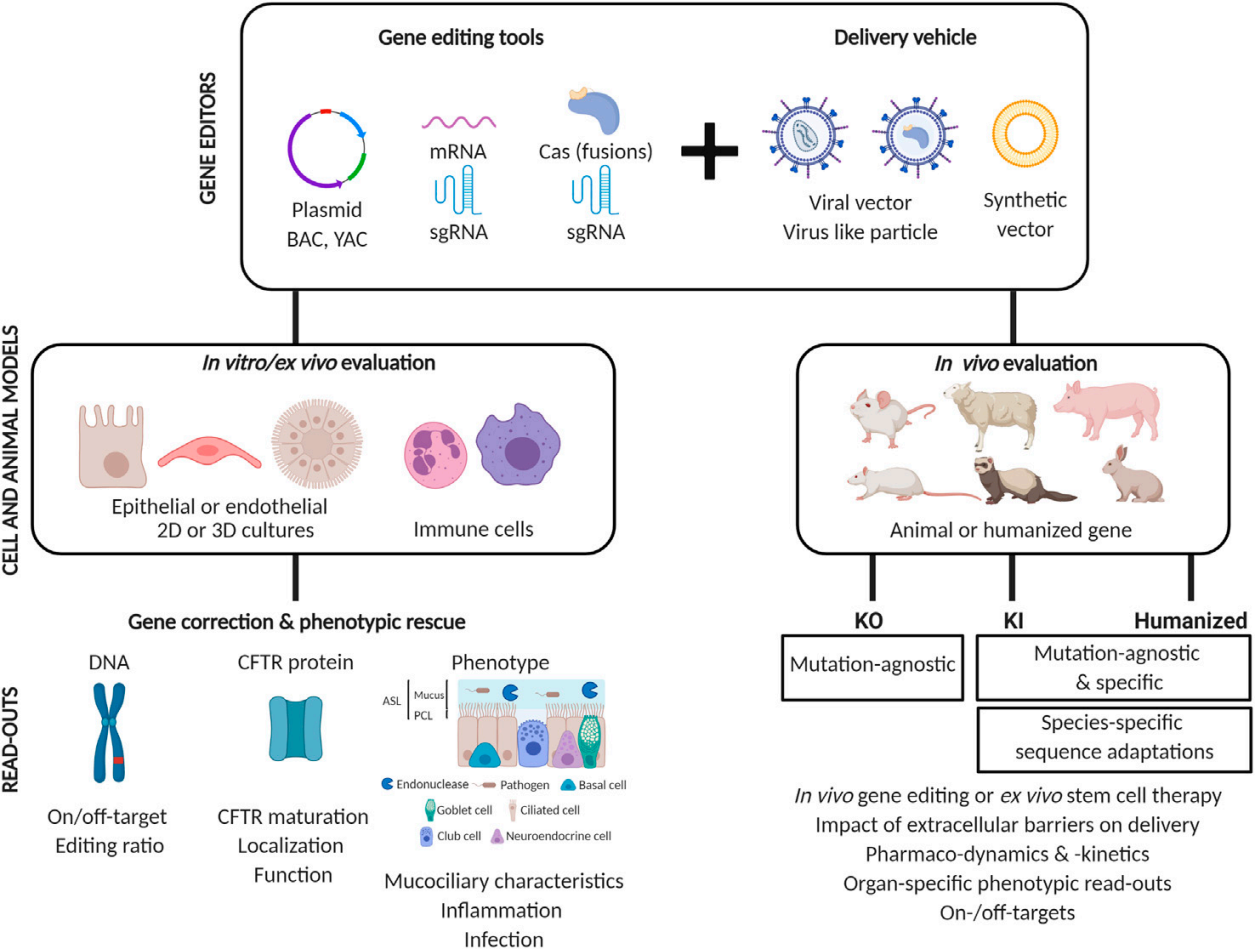
Gene editing strategies for CF from proof-of-concept to their translation into a therapy. Gene editing for CF consists of correcting mutations in *CFTR* either by an *ex vivo* stem cell therapy approach or by directly editing target cells *in vivo*. To transfer gene editors as nucleic acids or proteins into cells, it is likely necessary, particularly for the *in vivo* approach, to encapsulate them with a vector in order to protect them and facilitate their entry into target cells. For their clinical translation, *in vitro*/*ex vivo* and *in vivo* models are essential to determine the best formulation that allows efficient and safe gene editing. *In vitro* and *ex vivo* models allow evaluating gene editing efficacy at three levels: genomic, protein and physiological. Summarized, this starts by demonstrating a genetic correction with low off-targets, followed by a normalization of CFTR expression, folding and function, to end with a rescue of pathophysiological defects induced by mutant CFTR. Patient derived cell models furthermore allow developing personalized formulations for each patient. Animal models mimicking CF pathophysiology are a major asset to study the efficacy of the gene editing delivery vehicle in a clinically relevant environment. This, as extracellular barriers must be overcome such as thick and viscous mucus, pathogens or endonucleases to allow efficient gene transfer. Knock-out, knock-in and humanized models each have their specific advantages and limitations that should be considered for an *in vivo* evaluation. Abbreviations: ASL, airway surface liquid; BAC, bacterial artificial chromosome; Cas, CRISPR associated protein; KI, Knock-in models; KO, Knock-out models; PCL, periciliary liquid; sgRNA, single guide RNA; YAC, yeast artificial chromosome.

Delivery of the gene editing components into target cells also needs to be considered and will likely depend on the chosen strategy i.e., *in vivo* or *ex vivo*. Nuclease, guide (and donor template) should be expressed simultaneously in order to allow editing, but expression of the nuclease should, at the same time, be restricted in time so as to reduce the chance of off-targets. Gene editing components can be delivered either as plasmids, nuclease mRNA (with sgRNA) or RNP (reviewed in (Lino et al., 2018)). Equally important is the packaging of these components, so that they can efficiently reach and enter the target cells. Packaging can be either viral or non-viral (reviewed in (Maule et al., 2021)). Viral vectors are the most efficient in delivering cargo to target cells. As such, lentiviral vectors have been used to deliver Cas12a to intestinal organoids for correcting intronic splicing mutations (Maule et al., 2019). Depending on the viral vector, tropisms and immune responses vary, but transgene expression is usually medium to long-term, which is not preferred for nucleases. As an alternative, virus-like particles (VLPs) are developed to combine efficient delivery with transient transgene expression. VLPs mimic the protein structures of viruses but lack a viral genome, and can be filled with an RNA or protein cargo (reviewed in (Roldão et al., 2017)). Finally, non-viral vectors provide an alternative to deliver gene editing components to cells. They contain lipid, polymeric or inorganic particles which are less immunogenic than viral vectors and unlike viral vectors are not restricted in their packaging capacity, allowing to incorporate large cargos (reviewed in (Wilbie et al., 2019)). Non-viral nanoparticle-based delivery of PNA/DNA was used to correct mouse F508del in CF mice (McNeer et al., 2015). Independent of the gene therapy and delivery method, it is of the utmost importance that these are evaluated well preclinically in available cell and animal models. A summary of how the different cell and animal models of CF can be included in the preclinical evaluation of gene editing strategies is given in Figure 2.

## Discussion

The advent of highly effective CFTR modulator therapies for approximately 90% of CF patients has been a major game changer for PwCF. Besides that, it has transformed therapeutic development efforts to redirect their focus to the last 10% of patients carrying two minimal function mutations, not responsive to the market approved CFTR modulators developed by Vertex Pharmaceuticals. As many of these minimal function mutations are not rescuable by current small molecules, requiring minimal amounts of CFTR protein to further enhance their folding, function, or stability, other therapies need to be developed. Gene therapy, in the form of gene addition of a healthy copy of the human *CFTR* cDNA, has been investigated in more than 25 clinical trials since the early nineties. The most recent of these trials was based on the delivery of monthly doses of a liposome *CFTR* cDNA formulation, and led to a stabilization in lung function, providing hope that further improvements in gene therapy tools will increase the efficacy of such a therapy.

Correcting mutations at the endogenous *CFTR* locus heralds a new era of personalized medicine, which has increased hopes and expectations even more as this technology holds promise to cure the disease, if sufficient numbers of progenitor cells can be targeted in a safe manner. While many promising proof-of-concept studies have shown the feasibility to efficiently and safely repair human *CFTR* mutations in primary cell models, only limited studies have progressed toward an *in vivo* or *ex vivo* stem cell therapy approach. Indeed, there lie the biggest hurdles which require further investigation in order to progress from proof-of-concept to a real therapy. How to deliver in the presence of prominent extracellular barriers in the most affected organ in CF, the lung, remains one of the major hurdles to be tackled. Solutions being pursued in that direction are to treat earlier, before established chronic inflammation, infection and remodeling, or alternatively, to pretreat the lungs with mucolytic or hydrating solutions such as hypertonic saline or mannitol. While the holy grail would be to achieve a life-long correction, repeated doses will likely be necessary, which is only possible if immune responses to vector and gene editors can be controlled. The continuous developments in delivery vehicles, ranging from viral vectors to virus like particles and synthetic vectors provides a broad portfolio for balancing the targeting efficiency with a limited expression of the editing machinery to ensure transient and thus safer gene editing. It is clear that while primary cell models will allow to elucidate which mutations are correctable by the broad portfolio of gene editing tools, animal models will play a crucial role in answering the open questions on delivery, longevity of CFTR correction and how to deal with induced immune responses.

Finally, while single cell transcriptomic approaches have identified rare but high CFTR expressers in the lung, the ionocyte, it is not yet clear how important it will be to target specific cells within the lung, as each cell type plays an important role in protecting the airways from infection. Nevertheless, the prospect of gene editing is exciting and likely in the coming years, many of the open questions and currently identified hurdles will have obtained answers that further guide the development of a gene therapy for all PwCF.

## Glossary

ABE: adenine base editors
ALI: air-liquid interface
ASL: airway surface liquid
BAC: bacterial artificial chromosome
BALF: broncho-alveolar lavage fluid
Cas: CRISPR associated nuclease
CBE: cytosine base editors
CF: cystic fibrosis
CFBE: cystic fibrosis bronchial epithelium
CFF: cystic fibrosis foundation
CFTR: cystic fibrosis transmembrane conductance regulator
CGBE: C-to-G base editors
CRISPR: clustered regularly interspaced short palindromic repeats
DSB: double strand break
FRT: Fisher rat thyroid
GFP: green fluorescent protein
HA: hemagglutinin
HBE: human bronchial epithelium
hCFTR: human CFTR
HS-YFP: halide sensitive yellow fluorescent protein
HDR: homology directed repair
HEK: human embryonic kidney
iPSCs: induced pluripotent stem cells
Isc: short-circuit current
KI: knock-in
KO: knock-out
NBD: nucleotide binding domain
NHEJ: non-homologous end joining
NMD: nonsense-mediated mRNA decay
NPD: nasal potential difference
PAM: protospacer adjacent motif
PBS: primer binding site
PCL: periciliary liquid
pegRNA: prime editing gRNA
PNAs: peptide nucleic acids
PTCs: premature termination codon
PwCF: people with CF
rAAV: recombinant adeno-associated viral vector
RT: reverse transcriptase
SDF: small/short DNA fragment
sgRNA: single guide RNA
SMG: submucosal gland
ssODN: single strand oligodeoxynucleotide
TALE(N): transcription activator-like effector (nuclease)
UGI: uracil glycosylase inhibitor
UNG: uracil DNA glycosylase
WT: wild-type
YAC: yeast artificial chromosome
ZFN: Zinc finger nuclease
